# Practice of self-medication to manage oral health issues in a community setting of Nepal

**DOI:** 10.1186/s12903-025-05421-8

**Published:** 2025-01-07

**Authors:** Ashish Shrestha, Tarakant Bhagat, Santosh Kumari Agrawal, Ujwal Gautam, Naresh Prasad Joshi

**Affiliations:** 1 Department of Public Health Dentistry, BP Koirala Institute of Health Sciences, Dharan, Nepal; 2 Department of Public Health Dentistry, BP Koirala Institute of Health Sciences, Dharan, Nepal; 3 Department of Community Dentistry and Dental Public Health, Chitwan Medical College, Bharatpur, Nepal

**Keywords:** Community survey, Nepal, Oral health, Self-medication

## Abstract

**Background:**

Self-medication has been defined as the practice of self-diagnosis and medication use without seeking professional healthcare advice. Its prevalence for alleviating dental problems in Nepali communities has not been explored. This study was conducted to assess self-medication for oral health problems in a community setting in Nepal.

**Methods:**

A community-based cross-sectional study was conducted in two conveniently selected wards of Baraha Municipality of Sunsari district in August 2021 using a pre-validated questionnaire in the Nepali language. The prevalence of self-medication for oral health problems was assessed. The chi-square and Mann-Whitney U tests were used to check its association with demographic variables.

**Results:**

The prevalence of self-medication was found to be 43.3%. The most common health problem leading to self-medication was toothache (78.5%). Around half of the respondents (55.1%) replied achieving temporary relief after its use while around a quarter (25.1%) thought that self-medication was effective in taking care of their problems. Three-quarters of the respondents (75.7%) knew that they had to visit a dentist if their problems persisted. The annual family income was significantly associated with the practice of self-medication for oral health problems. (*p* = 0.013)

**Conclusion:**

Self-medication was found to be frequently utilized by the people of Baraha municipality with almost every second person with oral health problems reporting using it to solve their problems. This phenomenon is disproportionately seen in those with lower economic status.

**Clinical trial number:**

Not applicable.

## Introduction

Self-medication involves the use of medicinal products by the consumers to treat self-recognizable disorders or symptoms, or the intermittent or continued use of a medication prescribed by a physician for a chronic or recurring disease of symptoms. In practice, it also includes the use of medication by family members, especially where the treatment of children or the elderly is involved [1]. Self-medication can be a double-edged sword. It can be helpful in a country like Nepal, where healthcare facilities are not readily available, by alleviating acute symptoms and saving time. But it poses some significant problems. With improper medication or treatment methods, there is a possibility that the disease will worsen, the development of antimicrobial resistance, and, not to mention, adverse drug reactions [2].

Self-medication is a common phenomenon that transcends boundaries. A recent systematic review showed an alarming prevalence of self-medication in countries belonging to the WHO South-East Asian region [3]. Low socioeconomic status and inadequate health care along with poor access to these health services have been implicated as the reasons people opt to treat themselves [4]. Moreover, dental diseases have been cited as one of the most common reasons for self-medication [5, 6]. Although studies in the general public are rare, those conducted among patients visiting medical and dental hospitals have shown a high prevalence of self-medication in Nepal [7, 8]. 

Access to oral health services is an issue in developing countries such as Nepal. There are more than 4,000 dental practitioners registered on the medical council, which is approximately equal to meeting the needs of the country’s population around 30 million [9, 10]. However, the distribution of health services is not equitable in Nepal. A situation analysis done in 2017 showed that more than half of dentists worked in the capital alone [11]. Even now, many remote districts remain unfamiliar with the services of registered dentists. If people want dental services, they have to travel to the district headquarters, which inflicts direct and indirect cost on them. When treatment services are not available, people are naturally forced to find remedies for their maladies by themselves.

Dental services are hard to find, but dental problems are ubiquitous. Treating these problems requires trained manpower and well-organized facilities. The distribution of human resources and facilities to address these problems is unequal, especially in developing countries. At the same time, sociodemographic determinants also play a role in the utilization of health services. Fear of dental treatment has also been known as a factor for the under-utilization of dental services, even when they are available. Furthermore, in the context of the recent pandemic of COVID-19, dental services were mainly limited to the prescription of analgesics and antibiotics and the delay of routine dental care. These states of affairs point towards the possibility of self-medication being prevalent in rural communities. It seems likely that unnecessary consumption of antibiotics or analgesics may occur with possible side effects and possible antimicrobial resistance (AMR).

AMR is one of the top global public health and development threats. It is estimated that antibacterial resistance was directly responsible for 1.27 million global deaths in 2019 and contributed to 4.95 million deaths [12]. AMR is a problem for all countries at all levels of income, the spread of which recognizes no country borders [13]. Evidences suggest that self-medication might be contributing to increase in AMR in low and middle-income countries such as Nepal [14]. 

Self-medication can potentially increase and hasten AMR. At the same time, there is also a possibility that self-medication might play a beneficial role, especially in dire circumstances [1]. In the absence of any data on self-medication for dental problems in Nepal’s communities, this study was conducted to assess self-medication practice for oral health problems in a community setting in Nepal and the factors influencing the same.

## Methods

### Study design

A cross-sectional survey was carried out in two wards of Baraha Municipality in eastern Nepal from July to August 2021.

### Sample size

A sample size of 372 was calculated with a 95% confidence interval and a permissible error of 5% was considered for sample size adequacy based on a study in Uttar Pradesh, India [15]. 

### Study setting

**Fig. 1:**
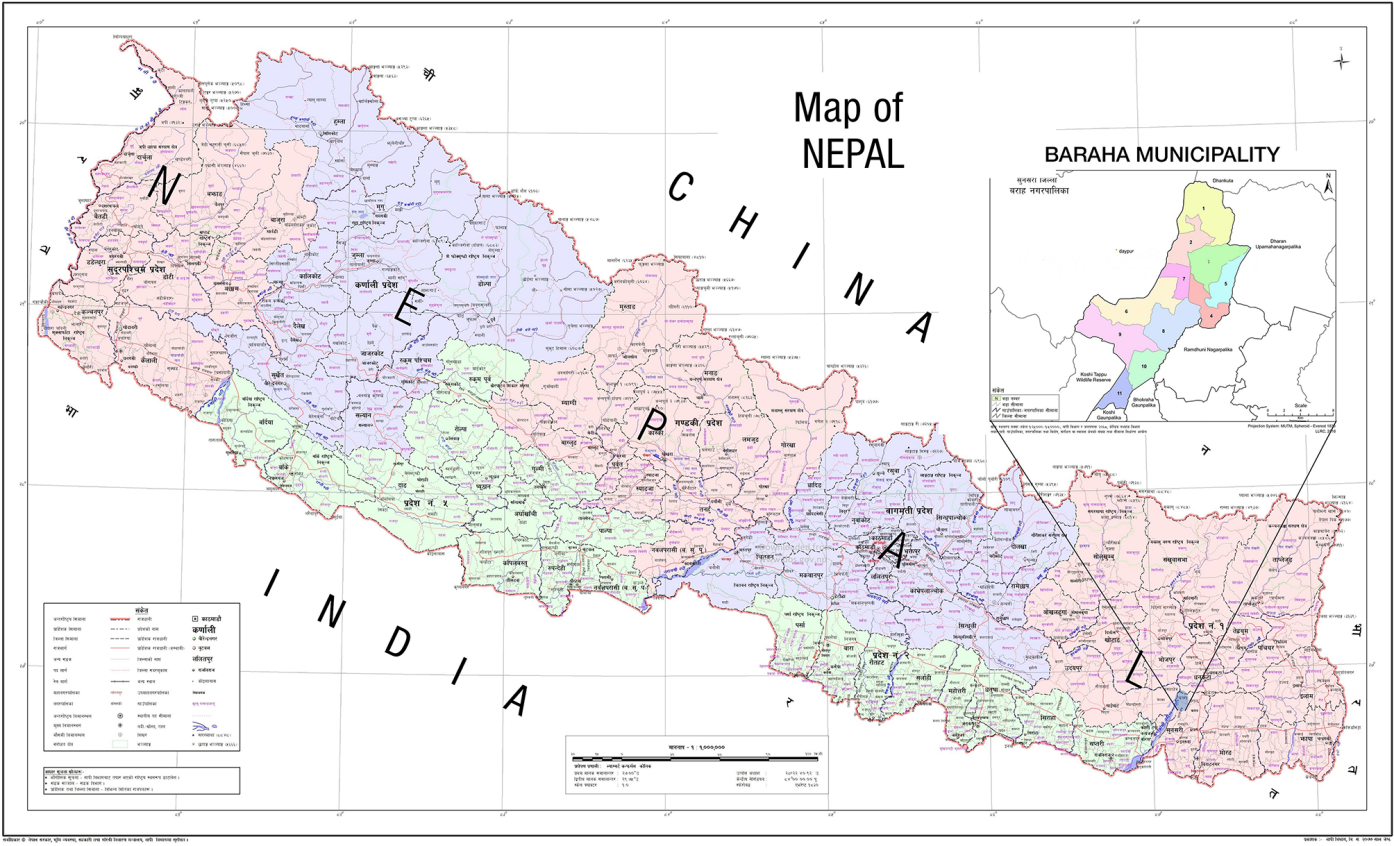
Map of Nepal showing wards of the municipality where the study was conducted

Nepal is administratively divided into seven provinces and 753 municipalities. The Baraha Municipality is located in the foothills of the *Chure mountains* with a population of around 100,000. The municipality is further divided into 11 administrative divisions called wards. Ward number 1 is home to the only municipal hospital where dental services are provided through an outreach centre of a tertiary hospital. The rest of the wards only have health posts, where basic medicines are provided free of cost but dental services are not available (Fig. 1).

#### Specific setting

Two wards of Baraha municipality were conveniently selected for this study. The households were systematically selected.

### Study participants

A participant was randomly selected from each household. Individuals aged 18 and older were eligible to participate. Those who were not able to answer for medical reasons or were not in a state to answer the survey questions during the house-to-house survey were excluded.

### Questionnaire and data collection

A pre-validated questionnaire [7] was used for data collection. The questionnaire included demographic variables such as age, sex, education, marital status, family income, social insurance status, and 10 questions related to self-medication. The questionnaire was administered by a single investigator after obtaining written informed consent. Data collection was carried out in August 2021.

#### Operational definitions

Duration of self-medication:

- Few days: when used for less than a week.
- Few weeks: When used for 1–2 weeks.
- Until the problem is over: When they continue practicing until their oral health problem is over or they seek professional treatment.

Types of self-medication:

- Analgesics: Medicines for pain relief available in pharmacies.
- Antibiotics: Type of antimicrobials available from pharmacies.
- Herbs: Locally practiced remedies such as a piece of clove put in the carious lesions, Neem, *Kattha* from Acacia.
- Others: Things like grains of rice, flowers claimed to be enchanted by local witch-doctors, anything that does not fall under the above definitions.

### Outcome measures

The main outcome measures were: (a) prevalence of self-medication for oral health problems and (b) factors affecting self-medication for oral health problems.

### Handling of data

Data were entered into Microsoft Excel 2016 and statistical analysis was performed using the Statistical Package for Social Sciences (SPSS) v. 11.5.

### Statistical analysis

Descriptive analysis was conducted for continuous and categorical data using mean, standard deviation, frequency, and proportion. Bivariate analysis was performed using the Mann-Whitney U test, Kruskal Wallis test and the Chi-square test to assess the association between the outcome variable of self-medication for oral health problems and sociodemographic variables. The level of significance was set at *p* < 0.05.

### Ethical approval

Ethical approval was obtained from the Institutional Review Committee, B P Koirala Institute of Health Sciences (Ref. 636/077/078-IRC). Informed consent was obtained from all the participants.

## Results

This community survey comprised 372 participants from the selected wards of the Baraha Municipality. The mean age (SD) of the participants was 40.5 (15.4) and the majority (57.8%) were females. The demographic characteristics of the participants are shown in Table 1.

**Table 1:** Demographic characteristics of participants from two wards of the Baraha Municipality, Sunsari, Nepal, July-August, 2021 (*n* = 372)

Variable	Frequency (proportion)
**Age** [(mean ± SD)]	40.58 ± 15.42
**Sample**	
Ward 1	176 (47.3)
Ward 2	196 (52.7)
**Gender**	
Male	157 (42.2)
Female	215(57.8)
**Marital status**	
Married	319 (85.8)
Unmarried	47 (12.6)
Widowed/separated	6 (1.6)
**Education** n (%)	
No formal education	66 (17.7)
Less than primary education	69 (18.5)
Primary education	68 (18.3)
Secondary education	120 (32.3)
Higher secondary education	37 (9.9)
Bachelor	10 (2.7)
Postgraduate degree	2 (0.5)
**Family monthly income** [mean ± SD]	23,650 ± 15,665
**Participation in Social insurance scheme**	185 (49.7)
**Occupation**	
Professional/technical/managerial	8
Clerical	2
Sales and services	108
Skilled manual	24
Unskilled manual	13
Agriculture	43
Student	30
Housemaker	126
Others	18

The occupation most frequently reported during this house-to-house survey was housemaker (33.8%). Almost one third of the participants were involved in sales and services (29%) (Table 1).

Health services were within a range of 2 KM for most of the participants. Only 81 participants (21.7%) reported being more than 2 km from a health center.

Out of 372 participants, 125 (33.6%) reported having no oral health problems. The prevalence of self-medication was found to be 43.3% (107/247).

Common reasons for self-medication included lack of time and money, previous experience with similar diseases, traditional beliefs, unavailability of doctors, the COVID-19 (Corona Virus Disease-19) pandemic, and fear of dentists. Toothache was by far the most common (78.5%) culprit in initiating self-medication. Other triggering factors were swelling, bleeding gums, halitosis, oral ulcers, dirty teeth, and food lodgment. More than half of self-medication users (55.1%) obtained temporary relief. Around a quarter of the participants thought self-medication was effective for their oral health problems. Around half of those who reported self-medication for oral health problems (44.8%) had used analgesics to relieve pain. Local herbs were also among the common self-medications. Participants also reported using clove oil, saltwater, and pepper to relieve their symptoms. Some atypical responses to the type of self-medication included ash, Khaini and *fukeko chamal* (enchanted rice grains; prescribed by a witch doctor). The most common source of self-medication was pharmacy (53.2%). Traditional homes were also used by around 20 per cent to avail of self-medication. The advice to use self-medication for oral health problems was received from relatives (36.4%) and friends (13%). A third of the users (30.8) said they were aware of the use of medications for their oral health problems. Finally, if their problem persists after self-medication, three in four people (75.7%) said that they would consult a dentist. A substantial number of respondents (11.2%) said that they would continue the same self-medication even if oral health problems persisted (Table 2).

**Table 2:** Details about self-medication practice for oral health problems in two wards of Baraha Municipality, Sunsari, Nepal, July-August 2021 (*n* = 107)

Questions	Frequency [*n* (%)]
**Reasons for self-medication**	
Lack of time	26 (24.3)
Lack of money	20 (18.7)
Previous experience	20 (18.7)
Traditional belief	20(18.7)
Unavailability of doctors	16(14.9)
COVID-19 pandemic	3 (2.8)
Fear of dentists	2 (1.8)
**Triggering factors**	
Toothache	84 (78.5)
Bleeding gums	8 (7.5)
Halitosis	2 (1.9)
Swelling	8 (7.5)
Others	5 (4.6)
**Duration of self-medication**	
Few days	76 (71)
Few weeks	4 (3.7)
Until the problem is over	27 (25.2)
**Types of self-medication**	
Analgesic	48 (44.8)
Antibiotic	6 (5.6)
Herbs	23 (21.5)
Others	30 (28.1)
**Source of acquiring self-medication**	
Pharmacy shop	57 (53.2)
Hospital pharmacy	11 (10.2)
Traditional home	21 (19.7)
Others	18 (16.9)
**Feeling after self-medication**	
Temporary relief	59 (55.1)
Effective	29 (27.1)
Useful in stressful situations	3(2.8)
Unsure	7(6.5)
Curative in nature	8 (7.5)
Cheaper option	1 (0.9)
**Advice for self-medication**	
Relatives	39 (36.4)
Friends	14 (13)
Personal knowledge	33 (30.8)
Pharmacist	17 (15.8)
Mass media	2 (1.8)
Social media	2 (1.8)
**Measures if the problem persists**	
Visit a dentist	81 (75.7)
Visit a medical practitioner	14 (13.1)
Continue with the same medication	12 (11.2)

Of all demographic variables, annual family income was found to be significantly associated with self-medication, with a greater proportion of individuals in lower income groups reporting its use. (*p* = 0.013) (Table 3).

**Table 3:** Association of self-medication for oral health problems with demographic variables problems of two wards of Baraha Municipality, Sunsari, Nepal, July-August, 2021 (*n* = 247)

Variable	Self-medication	No Self-medication	Total	*p*-value
**Age** (mean ± SD)	42.40 ± 15.42	38.58 ± 14.84	40.23 ± 15.18	0.059^#^
**Gender n (%)**				0.776*
Male	37 (44.6)	46 (55.4)	83	
Female	70 (42.7)	94 (57.3)	164	
**Marital status**				0.562*
Married	93 (44.1)	118 (55.9)	211	
Unmarried/widowed	14 (38.9)	22 (61.1)	36	
**Occupation**				0.205*
Employed	53 (47.7)	58 (52.3)	111	
Unemployed	54 (39.7)	82 (60.3)	136	
**Education**				0.224*
Cannot read and write	25 (51.0)	24 (49.0))	49	
Literate	82 (41.4)	116 (58.6)	198	
**Yearly family income [1$= 118.91 Nepalese Rupees (NRS)]**				**0.013** ^**‡**^
< 200,000 NRS	50 (49.0)	52 ((51.0)	102	
200,001-399,999 NRS	42 (47.7)	46 (52.3)	88	
≥ 400,000 NRS	15 (26.3)	42 (73.7)	57	
**Social insurance**				0.501*
Yes	52 (45.6)	62 (54.4)	133	
No	55 (41.4)	78 (58.6)	114	
**Distance from the nearest health center**				0.139*
< 1 KM	56 (48.3)	60 (51.7)	116	
≥ 1 KM	51 (38.9)	80 (61.1)	131	

#Mann Whitney U test

*Chi−square test

**‡** Kruskal Wallis test

Bold signifies statistical significance at *p*< 0.05

## Discussion

This study explored self-medication for oral health problems in a community setting in eastern Nepal. The prevalence of self-medication for oral health problems in this community was 43.3%, which is lower than that reported in India [15] (72%), and Cameroon [16] (67.8%). A higher prevalence has been reported in a developed country such as Saudi Arabia with a very high Human Development Index (HDI) [17]. A study carried out on dental patients visiting a hospital in Nepal [7] has shown a higher prevalence (62.6%) than that seen in our study. The study reports a prevalence comparable to that reported in Nigeria [18](42.5%). Burkina Faso [19], a landlocked country in western Africa, also has a similar prevalence of self-medication for oral health problems (48%). Our study suggests that family income is significantly related to the practice of self-medication. Differences in the prevalence of self-medication for oral health problems are also perhaps due to a myriad of factors such as sampling variability, differences in the availability of health services or the belief in traditional treatments [7]. 

Housemakers and students made up almost half of the study participants, as they were available at home during the house-to-house survey. There are considerably more women than men in Wards 1 and 2 of Baraha Municipality [20]. This is reflected in our study, as they make up more than 57% of the study population.

In the current study, the most common trigger factor that led to self-medication was toothache. Community-based surveys have shown that toothache is one of the common dental problems faced by people. A population-based survey in Iran [21] found that 55.1% of the participants have had toothache in the last 6 months. Similarly, a high prevalence of toothache has been reported in Brazil [22]. The use of analgesics to treat toothaches was found to be a common feature in our survey. Similar findings have been reported in Cameroon [16] and India [15]. On the surface, it looks like a benign phenomenon with occasional gastritis as a side effect but the use of analgesics to treat dental pain can lead to a rapid increase in dental problems. Simple reversible pulpitis can progress to periapical abscess or more severe clinical conditions before reaching a dentist. Surgical visits can save patients a lot of pain and suffering.

Saltwater was a common remedy reported in our study to alleviate their oral health problems. There is no scientific evidence to prove the efficiency of this method; clinicians often advise lukewarm saltwater rinse to their patients [23]. There is no prescribed dose of concentration or frequency of use, but it has not produced any side effects. Altman and Bland [24] have rightly said ‘The absence of evidence is not evidence of absence’.

In addition to toothache, other trigger factors leading to self-medication were bleeding gums, swelling, and halitosis. These are among the main complaints that patients present in dental outpatient departments. The duration of self-medication was a few days for most of the participants. It was intriguing to find that around a quarter of those using self-medication reported using it until the problem was over. Dental problems are seldom treated successfully with self-medication alone. E.g.; pain caused due to the progression of dental caries cannot be relieved with medication alone. A visit to a dentist for root canal treatment (or extraction, if the patient so desires) is mandatory. While self-medication may be acceptable as a temporary measure to manage pain in emergency situations, relying on it as a solution to address the underlying issue is inadvisable. Although symptoms may temporarily subside, the underlying condition is likely to recur with increased severity over time.

Apart from a few participants (around 10%), all others agreed that they had to see a dental practitioner to take care of their oral health problems. The reason they could not visit was lack of time and money, previous experience of treating similar diseases, traditional beliefs, and the inaccessibility of doctors. This reflects that self-medication is not only related to the inaccessibility of health services, but also has traditional and cultural determinants. Similar reasons have been cited in previous studies [15]. Regardless of the reasons they practised self-medication, the feeling they achieved was temporary relief.

The use of antibiotics as self-medication was only reported by a small proportion of people. However, in Nepal, the regulation of prescription drug sales is poor [25]. Therefore, the abuse of antibiotics may be more prevalent than that found in this study. Illiterate individuals may have failed to differentiate between analgesics and antibiotics while answering. The source of self-medication acquisition is pharmacy for more than half of users. This phenomenon of acquiring drugs from pharmacists without consulting a doctor is relatively common in Nepal [26]. In addition to side effects such as improper medication and incorrect treatment, it is contributing to increasing drug resistance. The medicines were also acquired from traditional homes. The herbs prescribed by people running traditional homes are based on no solid source of knowledge and cannot be trusted.

No differences were observed between male and female participants in their use of self-medication. Similar findings have been reported in a study in Cameroon [16]. Similarly, other sociodemographic variables such as marital status, occupation, education, and participation in social health insurance schemes did not show a significant association with self-medication. However, the practice of self-medication was found to have a strong association with family income. Interestingly, analgesics that have commonly been consumed without a prescription from a doctor are comparatively cheaper drugs. Both direct and indirect costs to obtain dental services may be a barrier for people visiting a dental hospital, especially in people with low family incomes [27]. 

There have been some studies in medical, dental and nursing students’ utilization of self-medication in Nepal [28–30]. Some studies also explore self-medication in community settings of Nepal as well [31–33]. But, to the best of the authors’ knowledge, this is the first house-to-house survey to explore self-medication for oral health problems in Nepal. It provides evidence of how people take care of their oral treatment needs. It also sheds light on the prominent issue of the unavailability of dental services, even to people living in municipalities of the country. If a patient receives antibiotics for the treatment of a periapical abscess from a health center, it is rational to think that they will take those drugs by themselves the next time a similar problem arises. Hospitals should provide their own specialist dental services. In the current scenario, only a handful of government hospitals in the country provide dental services in Nepal.

### Limitations

This study was not without limitations. The community-based survey was conducted during the day. The offices and transport services were open during this survey period, meaning that people who had certain jobs were not available at home for an interview. This may have compromised the representativeness of the sample as the wards were conveniently selected. Participants who could not read and write may not have been able to correctly report the medication they used. The difference between an analgesic and an antibiotic can sometimes be overwhelming, especially for those who have little education. This survey was conducted immediately after the COVID-19 lockdown was over. Hospital services were limited to the treatment of emergency services. People may have been using self-medication practices in the absence of accessible health services. At the same time, because of the COVID-19 pandemic, they might have considered oral health issues as minor problems and not reported them at all. Social desirability bias can be present while reporting about self-medication. In light of these limitations, our findings should be interpreted with caution. However, this study provides evidence of self-medication for oral health problems, most of which require a mandatory visit to dentists for surgical intervention.

## Conclusions

Our findings suggest that most people with low economic status use self-medication for a few days regardless of their distance to the nearest health service. They self-medicated with analgesics, herbs obtained from traditional houses, or warm saline water prepared by themselves. But commonly used drugs; analgesics do not cure dental diseases. Dental services should be made available and affordable to people, along with health education about the same. Although the findings suggest low antibiotic use, this survey may not have fathomed the bottom of the fabled iceberg. More research is warranted to determine the abuse of antibiotics through self-medication in communities.

## Data Availability

Data supporting the findings of this study are available from the corresponding author upon reasonable request.
